# EXOC3L2 rs597668 variant contributes to Alzheimer’s disease susceptibility in Asian population

**DOI:** 10.18632/oncotarget.15380

**Published:** 2017-02-16

**Authors:** Qing-Jian Wu, Shu-Yin Sun, Cheng-Jun Yan, Zi-Cui Cheng, Ming-Feng Yang, Zi-Fei Li, Hou-Wen Cheng, Ti-Kun Fang

**Affiliations:** ^1^ Department of Emergency, Jining No. 1 People's Hospital, Jining, Shandong, 272011, China; ^2^ Department of Neurology, Shandong University School of Medicine, Jinan, Shandong, 250012, China; ^3^ Key Lab of Cerebral Microcirculation in Universities of Shandong, Taishan Medical University, Taian, Shandong, 271000, China; ^4^ Department of Encephalopathy Rehabiliation Center, Taian Traditional Chinese Medical Hospital, Taian, Shandong, 271000, China

**Keywords:** Alzheimer's disease, genome-wide association studies, EXOC3L2, rs597668

## Abstract

Recent genome-wide association studies have established the association between EXOC3L2 rs597668 variant and Alzheimer's disease (AD) in European population. However, recent studies reported inconsistent results in Asian population. Here, we performed a systematic review and meta-analysis to evaluate the impact of rs597668 on AD risk in Asian population using a total of 8686 samples including 2855 cases and 5831 controls. Meanwhile, we selected 17,008 AD cases and 37,154 controls in European population to evaluate the potential heterogeneity between East Asian and European populations. In East Asian population, we identified no potential heterogeneity with *P*=0.31 and *I*^2^ = 15.8%. By meta-analysis, we identified positive association between rs597668 and AD risk with *P*=0.023, OR=0.93, 95% CI 0.87-0.99. We further found significant heterogeneity in pooled Asian and European populations with *P*<0.0001 and *I*^2^ = 87.7%. The meta-analysis indicated negative association with *P*=0.66, OR=0.97, 95% CI 0.85-1.11. In summary, all these findings indicate that rs597668 C allele is a risk factor for AD in European population with OR=1.18 and *P*=2.49E-13. However the rs597668 C allele played a protective role in AD with OR=0.93 and *P*=0.023 in East Asian population.

## INTRODUCTION

Alzheimer's disease (AD) is the most common dementia in elderly [1–3]. In recent several years, large-scale genome-wide association studies (GWAS) and next generation sequencing analysis have identified a number of AD susceptibility genes including CLU [4–8], CR1 [9–11], BIN1 [12–14], PICALM [15–18], CD2AP [13, 19], CD33 [20–21], ABCA7 [7–8, 11, 13–14, 22], TREM2 [23–24], MS4A4/MS4A6E [25–29], EPHA1 [25–29], and EXOC3L2 [25–30].

In these AD susceptibility genes above, a genetic variant rs597668 near EXOC3L2 was significantly associated with AD in European population with *P*=6.450E-09 [27]. The replication studies reported both positive and negative results [31]. Shang et al. conducted a meta-analysis by selecting 16 independent studies [31]. In overall datasets, Shang et al. reported significant association between rs597668 variant and AD [31]. In 2013, the largest GWAS further confirmed the significant association between rs597668 and AD with *P*=2.49E-13 in European population [32]. Shang et al. selected two studies in Asian population and 14 studies in European populations [31]. One study in Chinese population included 598 AD cases and 607 healthy controls [30]. Another study in Japanese population included 825 AD cases and 2933 controls [33]. However, both studies reported negative association between rs597668 and AD [33]. In above study, Shang et al. did not perform a subgroup analysis [31]. It is still unclear whether rs597668 is associated with AD in Asian population.

Here, we performed a systematic review and meta-analysis of the impact of rs597668 in AD in Asian population using a total of 8686 samples including 2855 cases and 5831 controls. Meanwhile, we selected 17,008 AD cases and 37,154 controls in European population to evaluate the potential heterogeneity between East Asian and European populations [32].

## RESULTS

### The characteristics of all the selected studies

In summary, we selected 12 articles in PubMed, Medline and CNKI databases. 3 articles were not conducted in Asian populations and then were removed. The remaining 9 articles were full-text reviewed, and 7 articles were excluded. In Google scholar database, we selected another 2 articles including 3 independent studies. In AlzGene database, we identified no article in Asian population. Finally, we selected 5 independent studies in Asian population and one study in European population as described in Table 1.

**Table 1:** The characteristics of six selected studies in this meta-analysis

Study	Population	Cases	Controls	Genotyping platform
Jiao 2015 [42]	Chinese	229	318	ABI 3730xl sequencer
Liu 2012 [30]	Chinese	571	607	ABI3130XL sequencer
Ohara 2012 [33]	Japanese	825	2933	Multiplex PCR-based Invader assay
Miyashita 2013 [43]	Japanese	891	844	Affymetrix GeneChip 6.0 and TaqMan
Miyashita 2013 [43]	Korean	339	1,129	Affymetrix GeneChip 6.0 and TaqMan
Lambert 2013 [32]	European	17,008	37,154	Imputation

### Meta-analysis in Asian population

**Figure 1:**
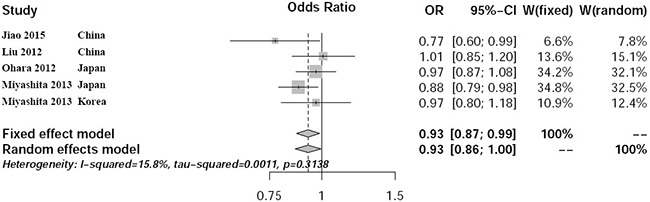
Forest plot about the meta-analysis in Asian population

**Figure 2:**
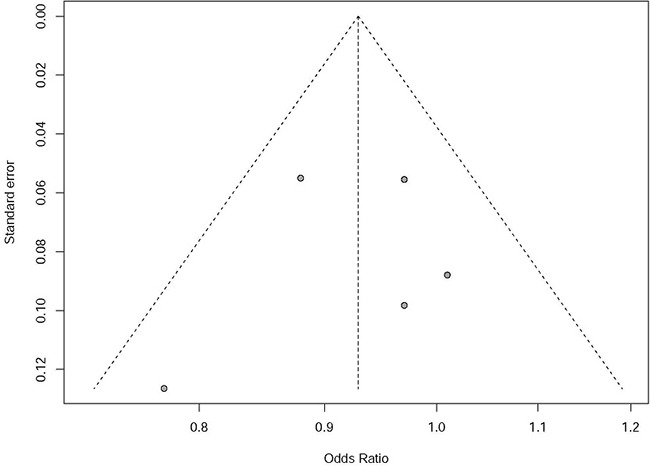
Publication bias analysis in Asian population

We identified no potential heterogeneity in Asian populations with *P*=0.31 and *I*^2^ = 15.8%. Meta-analysis using the fixed effect model showed significant association between rs597668 and AD risk with *P*=0.023, OR=0.93, 95% CI 0.87-0.99 (Figure 1). All the funnel plots are symmetrical inverted funnels (Figure 2). The statistical test further provides evidence of symmetry with *P*=0.78.

### Meta-analysis in Asian and European populations

**Figure 3:**
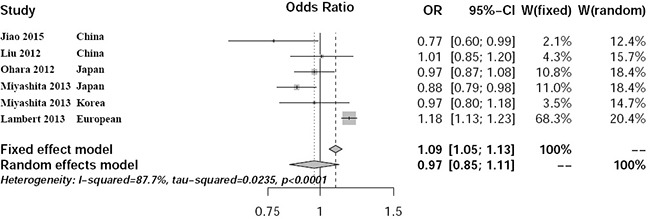
Forest plot about the meta-analysis in pooled Asian and European populations

**Figure 4:**
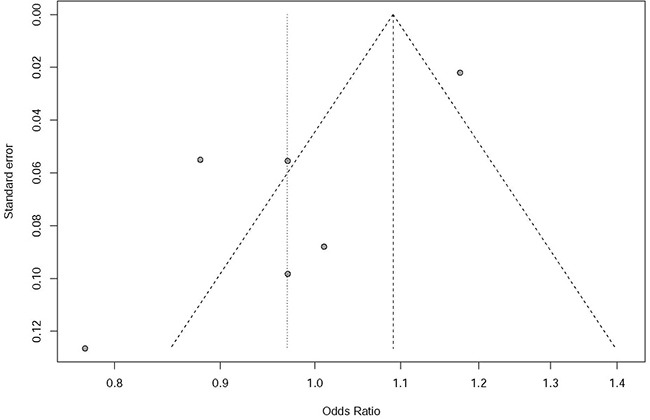
Publication bias analysis in pooled Asian and European populations

We identified significant heterogeneity in pooled Asian and European populations using C allele versus T allele model (*P*<0.0001, *I*^2^ = 87.7%). Meta-analysis with the random-effect model showed no association between rs597668 and AD risk with *P*=0.66, OR=0.97, 95% CI 0.85-1.11 (Figure 3). The funnel plot using all the selected studies is a symmetrical inverted funnel (Figure 4). The statistical test does not provide evidence of symmetry with *P*=0.034.

## DISCUSSION

Until now, the association between rs597668 and AD has been well established in European population. However inconsistent results have been reported in East Asian populations. Here, we conducted a systematic review and meta-analysis Asian population using a large-scale sample size including 8686 samples. We further compared the potential heterogeneity in Asian and European populations. In East Asian subgroup, we identified no potential heterogeneity with *P*=0.31 and *I*^2^ = 15.8%. By meta-analysis, we identified positive association between rs597668 and AD with *P*=0.023, OR=0.93, 95% CI 0.87-0.99. We further found significant heterogeneity in pooled Asian and European populations with *P*<0.0001 and *I*^2^ = 87.7%. The meta-analysis indicated negative association with *P*=0.66, OR=0.97, 95% CI 0.85-1.11.

In a previous longitudinal study, Schmidt et al. selected 40 AD cases, and identified rs597668 variant to be significantly associated with more aggressive disease courses [34]. The rs597668 C allele was associated with the risk of faster decline [34]. The largest GWAS showed that EXOC3L2 rs597668 C allele is a risk factor for AD in European population with OR=1.18 and *P*=2.49E-13 [32]. However, based on our findings above, the rs597668 C allele played a protective role in AD with OR=0.93 and *P*=0.023 in East Asian population.

In addition to the involvement of EXOC3L2 in AD risk, previous studies also evaluated the EXOC3L2 expression [35–36]. Wallgard et al. identified the up-regulation of the mouse *exoc3l2* homologue in brain vasculature [36]. Barkefors et al. identified that endothelial cells could express increased *exoc3l2* levels in developing blood vessels, and that the EXOC3L2 protein is associated with components of the exocyst complex [35].

In this submission process, we identified that there was no study to evaluate the association between rs597668 and AD using a meta-analysis in East Asian population. This is the first study investigating the association between rs597668 and AD by meta-analysis in East Asian population. We think that these findings may be very helpful for the future genetic studies. Following studies with large-scale sample size are also required to verify our findings.

## MATERIALS AND METHODS

In summary, we searched the PubMed, Medline, Chinese National Knowledge Infrastructure (CNKI), Google scholar and AlzGene databases to identify all possible studies with key words ‘Alzheimer's disease’, ‘EXOC3L2′ or “rs597668”. Meanwhile, we reviewed the reference list in the selected articles to manually identify all the additional relevant studies. We extracted the name of the first author; the year of publication; the population; the numbers of AD cases and controls; the OR with 95% CI. Cochran's Q test and I2=(Q−(k−1))Q×100% were selected to evaluate the potential heterogeneity[4–6, 10, 12, 15–17, 19, 21–24, 37–41]. The fixed effect model (Mantel-Haenszel) or random-effect model (DerSimonian-Laird) was used in meta-analysis [4–6, 10, 12, 15–17, 19, 21–24, 37–41]. Z test is used to calculate the significance of meta-analysis [4–6, 10, 12, 15–17, 19, 21–24, 37–41]. The potential publication bias was evaluated using both the funnel plot and statistical test method [4–6, 10, 12, 15–17, 19, 21–24, 37–41]. Here, we used R language to conduct all statistical tests. In all tests above, we define the significance threshold to be 0.05. The meta-analysis methods have been established in previous studies [4–6, 10, 12, 15–17, 19, 21–24, 37–41]. More detailed information is described in these above studies [4–6, 10, 12, 15–17, 19, 21–24, 37–41].
